# Assessment of a novel scanner-supported system for processing of child health and immunization data in Uganda

**DOI:** 10.1186/s12913-020-05242-1

**Published:** 2020-04-29

**Authors:** Annika Äijö, Ina Schäffner, Peter Waiswa, Rornald Muhumuza Kananura, Mesfin Kassaye Tessma, Claudia Hanson

**Affiliations:** 1 Department of Learning, Informatics, Management and Ethics (LIME), Karolinska Institutet, Tomtebodavägen 18, 17177 Solna, Sweden; 2 School of Public Health, Health Policy Planning and Management Department Makerere University, 7062 Kampala, Uganda; 3 Department of Global Public Health Sciences, Karolinska Institutet, Tomtebodavägen 18, 17177 Solna, Sweden; 4 Department of International Development, London School of Economics and Political Science, London, WC1E 7HT UK; 5 Department of Disease Control, London School of Hygiene and Tropical Medicine, London, WC1E 7HT UK

**Keywords:** Health information system, MyChild system, Immunization data, Low-resource setting, DHIS-2, Uganda

## Abstract

**Background:**

Electronic data capturing has the potential to improve data quality and user-friendliness compared to manually processed, paper-based documentation systems. The MyChild system uses an innovative approach to process immunization data by employing detachable vouchers integrated into a vaccination booklet which are then scanned and converted into individual-level health data. The aim was to evaluate the MyChild data capturing system by assessing the proportion of correctly processed vouchers and to compare the user-friendliness in term of time spent on documentation and health worker experiences with the standard health information system at health facilities in Uganda.

**Methods:**

We used a mixed method approach. Documented data were manually copied and compared to processed health records to calculate the proportion of correctly registered vouchers. To compare time spend on documentation we did a continuous observational time-motion study and analyzed data using a Mann-Whitney U test. Semi-structured interviews were conducted to assess health workers’ experiences and analyzed using conventional content analysis. Data was collected in 14 health facilities in two districts in Uganda using different systems.

**Results:**

The MyChild system processed 97% (224 of 231) of the vouchers correctly. Recording using the MyChild system increased time spend on documentation of vaccination follow-up visits by 24 s compared to the standard system (02:25 vs. 02:01 min/child, Mann-Whitney U = 6293, n_1_ = 115, n_2_ = 151, *p* < 0.001 two-tailed, Z = − 3.861, r = 0.186). However, high variance between health centers using the same health information system suggests that documentation time differences can be attributed to other factors than the way information was processed. Health workers perceived both health management information systems as predominantly functional and easy to use, while the MyChild system achieved a higher level of satisfaction.

**Conclusions:**

The MyChild system electronically processes individual-level immunization data correctly without increasing significantly time spent on recording and is appreciated by health providers making it a potential solution to overcome shortcomings of present paper-based health information systems in health centers.

## Background

Reliable health management information systems are vital to monitor vaccination and other health care services and facilitate appropriate resource allocation [1]. Strengthening health information systems is one of the WHO health systems building blocks [2] and an important aspect in pursuing the Sustainable Development Goals [3]. Systems with direct data entry at the point of service delivery and electronically captured individual-level data are ideal for timely, evidence-based decision making and health planning [4], and fundamental for health equity [5].

The presently implemented Health Management Information System of the Expanded Programme for Immunizations (HMIS EPI) in Uganda consists of a child health card, a child register, a child tally sheet, a health unit daily attendance summary, and monthly, quarterly and annual reports [6]. The monthly summary data are entered into the electronic District Health Information System (DHIS-2), which provides the Ministry of Health with monthly facility-level summaries. While the DHIS-2 presents an important innovation towards more timely availability key estimates, it still relies on paper-based summary sheets of only selected indicators. The compilation of the summary sheets is time-consuming, susceptible for calculation errors, and provides only aggregated data which does not allow for follow-up of individual patients [7, 8]. It also hinders the quantification of needs, causing stock outs and delayed routinisation of new vaccines [8]. Direct electronic data processing in facilities is proposed to overcome problems, but few solutions have been tested in relation to consumer-friendliness and reliability of data produced [9].

### The MyChild system

**Fig. 1:**
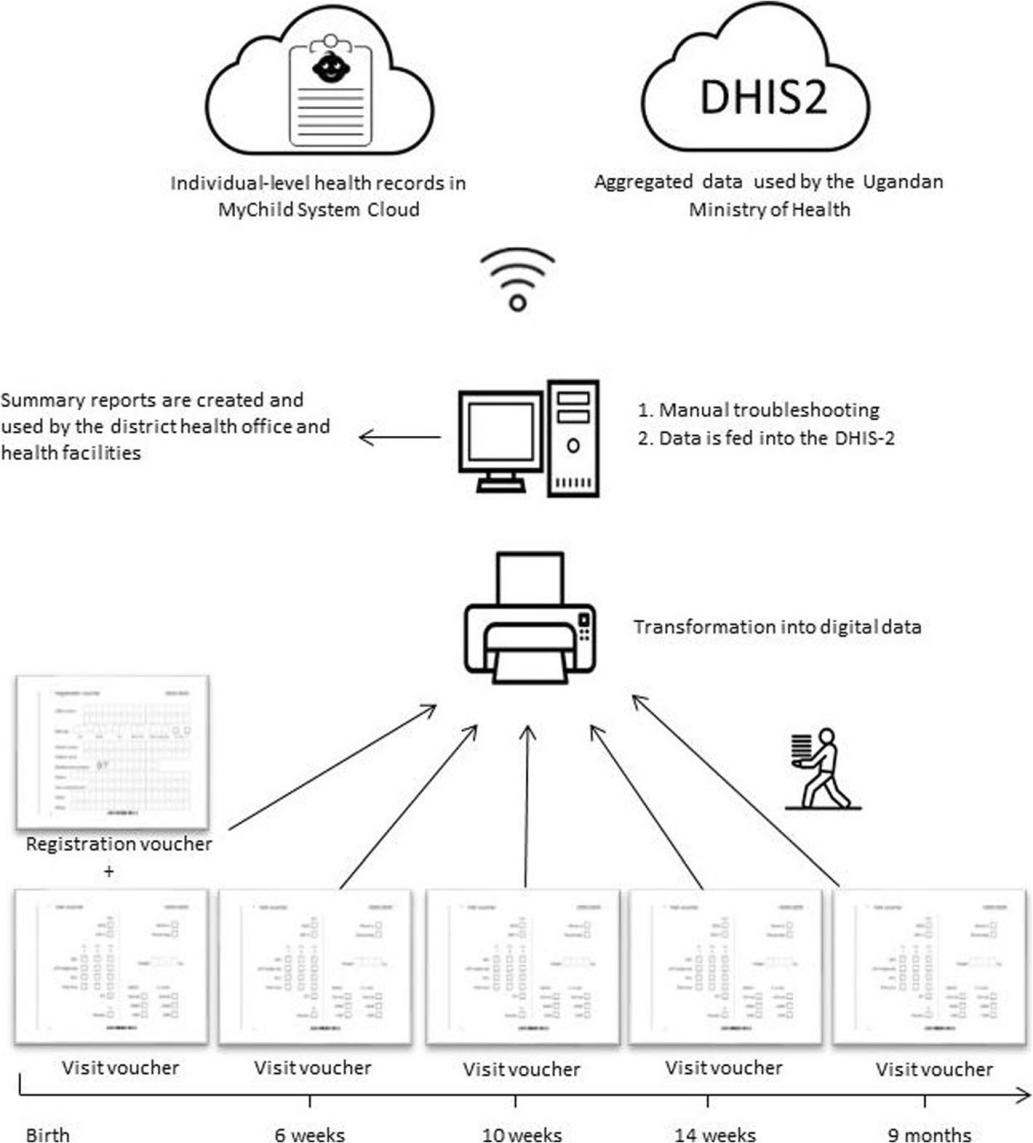
Visualization of the documentation procedure with MyChild system in Dokolo. At the first visit after birth, children are given a MyChild Card, a booklet with extractable vouchers on which information is recorded manually at the point of service. Vouchers are collected during the health service and brought to a central scanning station in the district about once a month where they are digitized with a Smart Paper Technology Engine. A quality assurance system identifies possible mistakes and unreadable handwriting. Detected issues are manually checked at the scanning station. After the quality assurance process, electronic health records for each child are compiled automatically via a child-specific identification code and stored in MyChild system cloud. The aggregated data is manually fed into the DHIS-2 system. The district health office receives summary reports through the DHIS-2 system and the MyChild System

The Shifo Foundation has developed a scanner supported data processing system; the MyChild system (see Fig. 1), to replace the paper-based HMIS EPI [10]. An additional file describes the kind of data captured with HMIS EPI and MyChild Card (see Additional file 1). The MyChild system automatically generates statistics of the collected data, and therefore health workers do not need to fill any summary sheets. The amendment only targets the primary documentation and the summary sheets, while further data processing with the DHIS-2 remains the same. The aim of the MyChild system is to register and follow-up on every child, to reduce administration efforts and to generate real-time data at individual and population level for decision makers [11]. Since the system replaces several registries, it may also reduce health workers’ time spent on documentation [11]. The MyChild system is currently used in three of 111 districts in Uganda [12]. Yet, it has never been independently evaluated.

## Methods

### Objectives

We aimed to evaluate the MyChild system by assessing the proportion of correctly processed vouchers and to compare the user-friendliness in terms of time spent on documentation and health worker experiences with the HMIS EPI at health facilities in Uganda.

### Study setting and selection of participants

The study was carried out in February and March 2017 in Dokolo and Bukedea, which both are rural districts with a population of about 200,000 people [13]. The two districts lie around 140 km away from each other and are located in the Central and Eastern parts of Uganda. Both districts have a typical pyramidal health structure and are semi-autonomous structures where data are needed for decision-making [14]. As it is standard with the present DHIS-2 systems, facility registers are maintained to document each patient contact. Monthly summary sheets of patient contacts and services provided are given to the district management team and data are entered into a web-based electronic application. The MyChild system was introduced in Dokolo in July 2016 and users had thus 9 months’ experience with the system. Bukedea was chosen as a comparison district as it is comparable to Dokolo in essential socio-demographic aspects [13].

Out of the total of 28 not-for-profit health centers (HC) that provided immunization and other preventive health services for children in these districts we randomly selected seven HCs from Dokolo and Bukedea respectively. We aimed to include HCs of equivalent grades of specialization (II, III, IV) in both districts. HCs II provide only basic services while HCs III and IV are increasingly specialized.

Study participants were health workers involved in providing preventive child health services. The population included nurses, nursing assistants, midwives, record assistants, members of village health teams and caretakers.

### Data collection process

Data was collected by following three different methods; data on MyChild Card vouchers were compared to the electronic health records, time spent on documentation was measured, and 14 health workers interviewed. The data collection methods are described below. AÄ and IS conducted all data collection. Prior to data collection, a pilot study was conducted in a randomly selected HC in Dokolo. A key observation was that different services were documented at different places, which made it impossible to measure child-specific documentation times. Thus the decision was to document the total time spend on documentation.

#### Assessment of data from MyChild card vouchers

To assess whether information entered on the MyChild Card vouchers was correctly processed, filled visit vouchers were copied manually at the end of each observation day at the HCs and later compared to the MyChild electronic health records. This information included tick boxes indicating provided services, a weight entry field and the child identification number. This process was performed at seven HCs in Dokolo by one researcher and double-checked by the second researcher. A voucher was considered as correctly processed if the electronic health record existed and was identical with the data indicated in the copy.

The sample size was calculated with an anticipated frequency of correctly processed vouchers of 95%, based on findings from comparable research [4]. Additionally, an assumed design effect of 1.5 resulted in a required sample size of 110 vouchers [15].

#### Observational study of time spent on documentation

To compare documentation times a comparative cross-sectional time motion study was conducted to measure the mean time spent on the documentation of preventive child health services per child. For this, the STAMP (Suggested Time And Motion Procedures) checklist was followed which considers relevant factors to ensure high-quality data collection and produce compatible and comparable results in time motion research [16]. An Android tablet with the Behavioral Observation Tool 3.4 [17] was used by one of two researchers (AÄ and IS) to track the time that health workers spent on documentation of follow-up visits. Tasks to be included in the time measurements were defined prior to data collection. The detailed descriptions of the included tasks are provided in an additional file (see Additional file 2).

The sample size was calculated using 80% power and 5% significance level with the software OpenEpi. The estimates for the standard deviation and the mean difference were based on expert recommendations. It was assumed that the time spent on documentation is 4 min shorter with the MyChild Card compared to the HMIS EPI. A standard deviation of 10 min was assumed for the MyChild Card and a standard deviation of 12 min for the HMIS EPI. This resulted in a sample size of 120 preventive child health service observations. Additionally, a design effect of 1.25 was assumed increasing the required sample size to 150 observations in each, intervention and comparison district [18].

#### Interviews with health care workers

A total of seven semi-structured interviews were conducted in each district, with one purposefully selected health worker at each HC. The interviews were conducted at the end of the day. Participants were informed prior to the interview that the aim of the study was to evaluate the documentation system. Interviews were led by one researcher (IS and AÄ) per HC. The interviews were conducted without the help of an interpreter, thus, only English speaking health care workers were selected. A closed room was preferred as a setting when available; otherwise, a quiet place outside the HC was chosen. Occasionally, nonparticipants were present during interviews. All interviews were recorded with a smartphone and later transcribed.

An interview protocol was developed based on the validated health information system monitor questionnaire [19, 20] and structured into four main themes: general experiences, problems, use of data and suggestions for improvement of the system in place. For each theme, probing questions were prepared and used if aspects were not mentioned by interviewees themselves.

### Data analysis

All statistical analyses were performed using SPSS Statistics 23 and the level of significance was specified at 5%. Regarding the proportion of correctly processed vouchers our null hypothesis was that 90% of the vouchers were correctly processed. The alternative hypothesis was that the proportion of correctly processed vouchers is different from 90% (two-sided). A z-test for a single proportion was performed.

In order to analyse the differences between times spent on documentation in the two districts a Mann-Whitney U test was performed due to deviation of normality. For sensitivity analyses we compared differences between health facilities using one way ANOVA. We employed a Levine’s test to check the assumption of equality of variance for the ANOVA. The partial eta-squared (η2) was used to evaluate effect size. Since the overall ANOVA test was significant, we performed further analysis using a Gomes Howell posthoc pairwise comparison. We used Gomes Howell since there was violation of the equality of variance assumption. Datasets from two HCs II were excluded because not all documents were used respectively because no scale was available during the health services and time-measurement data from one HC III was lost due to a technical error.

To analyse the interviews we used content analysis with an inductive approach to minimise the impact of researchers’ bias and prepossessions in the analysis process [21]. The interviews were transcribed by one and the transcripts checked by the other author to ensure correctness. After familiarizing with the content and highlighting key concepts, all data was coded and condensed by focusing on meaning. A common coding scheme was developed to group codes into categories and sub-categories. Finally, findings were compared between the districts. Codes that were identified in both districts were counted to enable an objective analysis of the interviews.

## Results

At HCs in Bukedea, two to three health workers provided the services, while in Dokolo it varied between three and seven. Characteristics of interviewees, total time of observations per facility and additional factors impacting the time motion study are listed in Table 1. The organization and procedure of immunization sessions is described in an additional file (see Additional file 3).

**Table 1:** Characteristics of the included facilities and the interviewed health workers

Health centre	System in place	Total number of staff giving child health services	Number of staff document- ting	Service duration (hh:mm)		Interviewee			
					Number of children	Age	Sex	Years worked at HC	Observation of factors impacting time motion study
HC1	MyChild system	4	2	02:35	24	58	M	10	Weight not plotted
HC2	MyChild system	7	6	02:37	33	40	M	10	
HC3	MyChild system	5	3	02:12	30	26	M	3	
HC4	MyChild system	7	4	02:48	33	42	M	5	
HC5	MyChild system	3	2	01:41	20	50	M	10	
HC6^a^	MyChild system	5	4	01:58	40	45	M	15	No scale at the facility
HC7	MyChild system	6	1	01:55	11	29	M	3	Weight not plotted
HC8	HMIS EPI	2	2	02:16	12	28	F	3	
HC9	HMIS EPI	3	2	01:36	26	58	M	10	
HC10	HMIS EPI	3	2	03:12	28	60	M	7	
HC11	HMIS EPI	2	2	02:50	33	36	F	14	Weight sometimes plotted
HC12^a^	HMIS EPI	3	2	04:36	102	40	M	9	No scale at the facility and Child register barely used
HC13	HMIS EPI	2	2	02:24	16	42	M	14	
HC14^a^	HMIS EPI	2	1			63	F	2	Time motion data missing

^a^ Datasets were excluded due to non-conformity with inclusion criteria or lost due to a technical error

### Correctly processed vouchers

All copied vouchers were transferred into digital health records. The proportion of correctly processed vouchers by the MyChild system was 97% (224 of 231) (95% Confidence interval 94–99%, z = 3.546, p < 0.001) (Table 2).

**Table 2:** Proportion of correctly processed vouchers and number of weight field entries captured with the MyChild system

	N	%
Visit vouchers analysed	231	
Visit vouchers missing	0	
Visit vouchers correctly processed	224	97.0
Weight fields analysed	130	100.0
Weight fields correctly processed	130	100.0

On the 231 vouchers 636 tick boxes and 130 weight entry fields were to be filled. Each voucher displays 24 possible tick boxes and one weight entry field. The appropriate number of tick boxes to be filled depends on the services provided. A total of 101 weights fields were not filled because the facilities did not have weighing scales. All weight entry fields were processed without error. Seven vouchers were identified as false due to following errors: one tick mark on a voucher was not recognised; one was crossed out but registered as marked; and on five of the vouchers nine ticks were added after the observations at the health centre.

Further observations during the assessment of correctly processed vouchers were that for five children several vouchers were filled and scanned. In two of these cases the information on the duplicate vouchers was identical; in three cases it was contradicting each other.

### Time spent on documentation

**Fig. 2:**
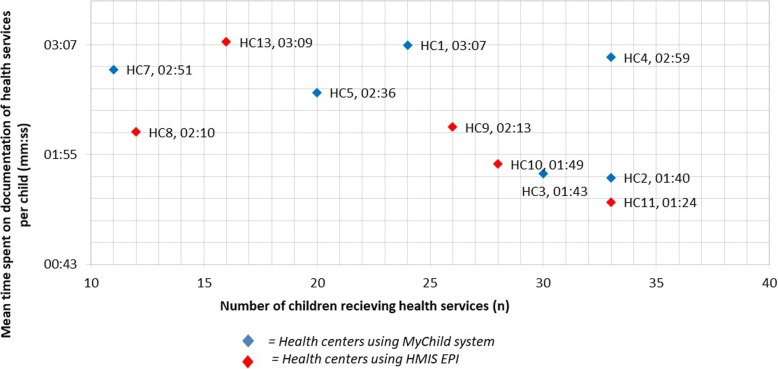
Mean time spent on documentation of preventive child health services per facility with MyChild system and HMIS EPI

On average, a mean time of 02:25 min was spent on documentation of follow-up visits in HCs using the MyChild system and 02:01 min in HCs using the HMIS EPI, giving a difference of 24 s between the two districts (see Table 3). The distribution differed statistically (median MyChild system = 02:24, median HMIS EPI = 01:49, Mann-Whitney U = 6293, n_1_ = 115, n_2_ = 151, *p* < 0.001 two-tailed, Z = − 3.861, r = 0.186). The mean time spent on documentation per HC is shown in Fig. 2.

**Table 3:** Mean, median and mean difference in time spent on documentation of preventive child health services at follow-up visit with MyChild system and HMIS EPI

	MyChild system		HMIS EPI		Mean difference
	mm:ss	n	mm:ss	n	
Mean time	02:25	151	02:01	115	00:24
Median time	02:24	151	01:49	115	00:41

One-way ANOVA revealed statistically significant mean differences in mean time spent on documentation between the HC [F (10, 255) = 22.1, *p* < .001, partial η2 = .46]. Results of post hoc pairwise comparison using Gomes Howell demonstrated that there were significant mean differences between the health facilities. Between the two districts, 16 pairwise combinations showed statistically significant differences in the mean time spent on documentation (data not shown). However, we did not observe statistically significant mean differences in 14 combinations. Noteworthy, comparison even showed significant differences within the districts.

### Health worker experiences

From the data collected during the interviews we identified six categories: Opportunities, Challenges, Suggestions for improvement, Use of data, Other challenges and Organisation of services. The corresponding sub-categories, codes and at how many HC they were mentioned are presented in an additional file (see Additional file 4).

#### MyChild system

All interviewees in Dokolo expressed contentment with the MyChild Card, in some cases very strongly. One health worker who had been working with vaccination services for over 10 years said: *“MyChild Card why I loved it’s not only because it’s just nice. / … / it has everything”*. Interviewees in Dokolo found it useful, easy to use, easy to find information in the card and fast to fill it. They said furthermore that it contains all necessary information, and they liked the possibility to get information from the district health office in cases where the card is lost. One interviewee said: “*why I loved it is like: once the card gets lost and then you have kept the other proof of ID number, it actually helps to know the child’s vaccine for next, for next visit”.* Two health worker said that they like the size of the MyChild Card, one of them said: *“it’s even portable, it’s unlike the other one, that used to be very big, and makes handling very difficult”.* Another comment in Dokolo about the previous system was that particularly the use of the child register was difficult. Many health workers reported difficulties filling in the MyChild Card when it was newly introduced and would have appreciated more training on it.

#### HMIS EPI

Health workers in Bukedea did not approve the health information management system as strongly as in Dokolo but they were overall satisfied with the HMIS EPI; one health worker said: “A*s it is now, there is nothing that deserves change / … / because all those documents are okay”.* Interviewees found the system easy to use, and that it was easy to find information in it, as one health worker with 10 years of experience described as follows: “*It is very easy to find the right page, because we go by date by date, we just follow date by date, it is very easy”.* Only one health worker felt that it was tiresome to look for children in the child register. Health workers generally experienced the time spent on documentation as acceptable but one mentioned that parts of it are skipped when they are too busy. Opinions about the compilation of monthly summaries were diverse; three felt that it is a lot of work and difficult to compile the monthly summaries while others reported the contrary. Although most health workers using the HMIS EPI liked the current follow-up method of defaulters, two health workers said that they did not use it because it is time consuming. The most common challenges health workers experience in both districts are stock-outs of supplies, lack of materials, no transport for outreaches and too low salaries. Furthermore, it was mentioned as challenging in both systems if caretakers move between HCs.

## Discussion

The MyChild system processed 97% (224 of 231) of the vouchers correctly. Recording using the MyChild system increased time spent on documentation of vaccination follow-up visits marginally by 24 s compared to the standard system (02:25 vs. 02:01 min/child). Overall, health workers in both districts were positive towards the health information system. However, users of the MyChild system expressed satisfaction in a stronger way than users of the HMIS EPI.

The study result, that 97% of the vouchers were correctly processed, indicates that the MyChild system processed ticks and hand written numbers on the vouchers well and generates reliable health records. Results from similar studies about the accuracy of scanning health records vary largely, between 57.5 and 99.8% [4, 22]. Scanning health records to digitalise health data is a relatively new technique and software for reading scanned data is constantly improving.

Our qualitative study indicated good acceptability of the MyChild system among the health providers. They liked the MyChild Card better than the HMIS EPI Child Health Card that they used earlier.

The difference of 24 s in documentation time between the MyChild system and the HMIS EPI at the point of service is marginal and its practical significance questionable. High variance between HCs within the districts indicate that other factors beyond the data processing systems were reasons for these differences, e.g. some health workers write faster than others and some HCs had significantly longer queues than others. It is to note, that most electronic information systems used in health do not save health providers time [23]. Also, it needs to be emphasised that the times measured only included documentation activities at the point of service. Documentation activities taking place off service site, such as scanning, manual data inputs and monthly summaries were not part of the study. This reduces the extent to which the health management information systems can be compared regarding documentation efforts. We identified only one published study from the United Kingdom which supported our finding that digitalising health data using scanning technologies does not put any major operational burden on health providers [24].

The value of the MyChild system is that it generates electronic health records on an individual level which allows the analysis of who has received immunization services in terms of place and patient characteristics. Moreover, the system replaces several summary sheets, the tally sheet and the child register. It is also well adapted to the resource constraints as the documentation at site is paper-based and does not depend on stable power supply. The system also includes vouchers for stock management which allows health workers to inform the district health office regarding stock-outs and other problems in a systematic way. The MyChild system has therefore the potential to enable better planning and development of immunization service delivery.

Most innovations of electronic health records demand reliable technical support systems [24]. A systematic review published in 2012 indicated high costs of procurement and maintenance, poor network infrastructure and lack of comfort among health workers with electronic medical records [9]. Such findings caution the move toward digitalising health data at the point of care in view of the large health worker shortages like in Sub-Saharan Africa [25]. Since the MyChild system is paper-based at point of care, it avoids these potential issues.

One shortcoming of the MyChild system is that if the child health cards are out of stock, then there are no other way to record. When this happened in Bukedea where they used the HMIS EPI, they could still write in the child health book, the tally sheet and use any booklet as a child health card. Another challenge with MyChild system could be the need of transportation of the vouchers from the HCs to the scanning station at the district health office. This did not pose any problems in Dokolo, however, the reliability of the process of moving the vouchers to a scanning station will need testing in any new setting. The HMIS EPI tally sheet enabled the health providers to easily see every day how many children had been vaccinated. This was not possible when using the MyChild system since the summaries were generated at the district health office or at the hospital.

The advantages and disadvantages of this system should be viewed also against the large potential which electronic health data bear. Mobile phone reminders that are automatically sent by the MyChild System to mothers of children who were missed for scheduled vaccination sessions are only one opportunity [26]. Several studies have indicated the usefulness of such digital innovations in HIV care delivery [27, 28].

In view of our results indicating that 97% of data correctly processed without substantially increasing the health workers time spent on recording, we believe that the system should be further developed and tested including efficiency and cost-effectiveness covering the whole continuum of maternal and newborn care and at larger scale.

### Methodological considerations

A strength of this study was the inclusion of seven HCs of different levels, corresponding to half of the HCs in both districts. Further, the number of children receiving preventive health services observed at HCs was high. Being present at HCs throughout the whole day allowed the researchers to get to know health workers and win their willingness to answering interview questions openly. The immediate copying of documents after services allowed an assessment of whether vouchers were lost during transport or at the scanning station. Being two researchers allowed for double checking and thus minimised the number of mistakes and extent of bias during data collection and analysis. To minimise the effect of inter-researcher variability in the time motion study the activities included and excluded from timing were precisely defined.

An aspect that was not practically feasible to assess was whether fields in the documents that were supposed to be filled actually were filled. This might have impacted the results of the time motion study in the two districts disproportionally. Another limitation was that communication was included as a documentation activity since it was sometimes done in parallel to writing. It was also a limitation that the vouchers were copied instead of directly compared to the database. The fact that the study was not blinded might have led to the Hawthorne effect; the awareness about being observed could have caused health workers to document particularly accurately or particularly quickly. The absence of a translator during the interviews was a limitation since not all interviewees were able to express themselves sufficiently since English was not their first language. Furthermore, a selection bias was introduced by excluding individuals who could not speak English. Another limitation was that non-participants listened to some of the interviews since the interviewee may have felt constrained in how openly they could answer the questions.

Our study did not investigate into the scalability of the system [29], but the responses from the health providers indicate general acceptance. Other aspects which will need more research are the costs and cost-effectiveness of the system compared to the traditional way of operations. It is also unclear whether the positive experience is transferable to other areas such as antenatal or intrapartum care where much more complex data will need to be processes. The possible negative correlation between the numbers of children treated per immunisation session with the mean time spent on documentation per child should also be analysed.

## Conclusions

The MyChild system shows potential as a purposeful and user-friendly health management information system for processing preventive child health data in Uganda. The system processed 97% of the vouchers correctly. Health care workers liked the MyChild Card and found it easy to use. Further studies should examine the intervention at a large scale and its economic effectiveness.

## Supplementary information

**Additional file 1.** Information captured with HMIS EPI and MyChild Card. A table that shows what kind of data is captured with HMIS EPI and MyChild Card.

**Additional file 2.** Activities included in time motion study. A detailed description of what is defined as documentation activity in the time measurements.

**Additional file 3.** Organization of vaccination sessions in Northern Uganda. A description of how a typical health service session was organised.

**Additional file 4.** Code scheme for interview analysis. Interview results are summarized in a table including all sub-categories and codes.

**Additional file 5.** Time motion data. Raw data for time-motion analysis.

**Additional file 6.** Scanned voucher data. Data on all vouchers that were observed, scanned and analysed. Furthermore details about problems are described.

## Data Availability

The datasets supporting the conclusions of this article are included within the article and its additional files (see Additional files 5 and 6).

## Abbreviations

DHIS: District Health Information System
HC: Health Center
HMIS EPI: Health Management Information System of the Expanded Programme for Immunizations
