# Supplementary material for: Assessment of a novel scanner-supported system for processing of child health and immunization data in Uganda
Source: BMC Health Serv Res. 2020 Apr 29;20:367. doi: 10.1186/s12913-020-05242-1 (PMC7191783; doi:10.1186/s12913-020-05242-1)
Supplement: Supplementary file 1 — Additional file 1. Information captured with HMIS EPI and MyChild Card. A table that shows what kind of data is captured with HMIS EPI and MyChild Card. [file 12913_2020_5242_MOESM1_ESM.docx]

**Information captured with HMIS EPI and MyChild Card**
