# Supplementary material for: Assessment of a novel scanner-supported system for processing of child health and immunization data in Uganda
Source: BMC Health Serv Res. 2020 Apr 29;20:367. doi: 10.1186/s12913-020-05242-1 (PMC7191783; doi:10.1186/s12913-020-05242-1)
Supplement: Supplementary file 2 — Additional file 2. Activities included in time motion study. A detailed description of what is defined as documentation activity in the time measurements. [file 12913_2020_5242_MOESM2_ESM.docx]

**Activities included in time motion study**

**Start of time measuring**

Time measuring started when health workers started any activity defined as documentation activity as listed below in the MyChild Card, child health card, child register, tally sheet or soft cover notebook that is used when no child health card is available.

Activities included in the time measurement:

- Opening, flipping pages in order to find right page to document on
- Documenting information in any of the above mentioned
- Asking caretaker for information to document
- Looking in the calendar for next date of visit
- Discussing questions about documentation with colleagues

**End of time measuring**

Time measuring ended when any of the documentation activities stopped.

Activities excluded from the time measurement:

- Taking up card, book or tally sheet
- Waiting for mom to come to the desk
- Filling tally sheet in Dokolo
- Summarizing tally sheet in Bukedea
- Filling of monthly, quarterly and annual reports in Dokolo
- Filing session voucher in Dokolo
