# Supplementary material for: Assessment of a novel scanner-supported system for processing of child health and immunization data in Uganda
Source: BMC Health Serv Res. 2020 Apr 29;20:367. doi: 10.1186/s12913-020-05242-1 (PMC7191783; doi:10.1186/s12913-020-05242-1)
Supplement: Supplementary file 3 — Additional file 3. Organization of vaccination sessions in Northern Uganda. A description of how a typical health service session was organised. [file 12913_2020_5242_MOESM3_ESM.docx]

**Organization of vaccination sessions in Northern Uganda**

Public health care delivery structure in Uganda is decentralized and organized by national, regional and general hospitals, health centers type IV, III, II and I who have different grades of specialization. Health centers type I have no static location but comprise community outreaches done by village health teams.


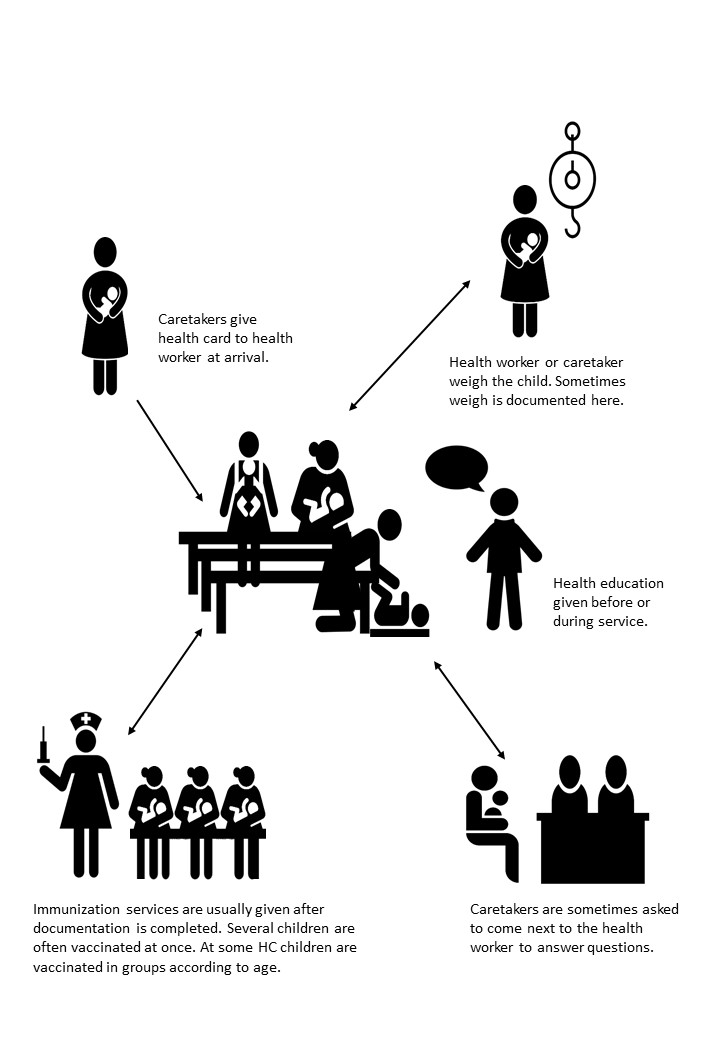
A key observation during data collection in two districts in Uganda was that the organization of delivery of vaccination services varied significantly between health centers. No correlation was seen between health centers within the same district or of the same specialization grade. The illustration below shows typical elements of a vaccination service: handing over of child health card, weight measurement, health education, individual consultation, immunization. Not all elements were performed at all centers and the order in which they were provided was different between and in some cases even within health centers.

Immunization sessions took either place inside the health centers, on the front porch or outside in the yard under a tree or other shady place. Health workers set up some chairs for themselves to sit on and a table with the vaccination equipment. Caretakers either sat on benches or chairs if available or on the ground while waiting. A scale was hung up in a door frame, branch of a tree or similar. Not all health centers had a scale and few had infant scales available.

Vaccination sessions involved between one and six health workers at the same time. Most centers had a specific starting time for the immunization sessions at which caretakers were expected to arrive. At all centers, caretakers were asked to hand in their child’s health card at arrival. Often, cards were put in a pile in the order they were handed in and later used as a queuing system. Health education was most commonly given in the beginning of the session to the group of caretakers that were present by that time. Information given usually included the importance of breast feeding, vaccination and contraception. A few health centers used educational material like painted illustrations or samples of for e.g. condoms. Very rarely, caretakers were given individual health counselling and the opportunity to ask questions in a place that allowed for confidentiality. In some cases, while one of the care takers gave the education talk, the others started to look at the child health cards. Sometimes, the child health cards were filled in already at this point. After the health education, commonly, health workers called up child names whose caretakers came to the front and were asked some questions about the child while information was entered in the health card. This took longer time for infants that came to their first immunization session and where all basic information needed to be entered. The process of weight taking differed a lot. In some health centers, health workers weighed the children themselves while entering information into the cards, in some caretakers were asked to go and weigh their child and come back to the health worker who wrote down the information. In other cases, caretakers were asked to weigh their child at any point while waiting for immunization and ask one of the health workers to write it down either later while vaccinating or directly. The latter often gave a less organized impression since caretakers communicated across longer distances and health workers had to find the right child card. In individual cases researchers observed that weights were entered incorrectly or that multiple vouchers were filled for the same child with conflicting information. The immunization itself was usually done by the health worker asking one caretaker at the time to bring their child to the front, sit on a chair and hold it while immunization is administered. This was commonly done directly after filling of health card. In many cases though, these tasks were performed by different health workers. For example, one to three health workers took cards from a pile, filled in which vaccine was due, when to come back for follow up and what the child weighed for which they asked towards the group of caretakers. Then the card was put on a new pile from which one or several other health workers took them, called up the caretaker and their child and administered the vaccine. At some facilities they completed most of the documentation before they started the immunization. The reason for this was that they tried to keep the vaccines cold by keeping them in a refrigerator a little longer. At one health center, health cards were sorted by number of visit and thereby which vaccines were due. One group of caretakers at the time was asked to sit on a long bench and make free a relevant part of the child’s skin. One health worker administered vaccines while the others prepared syringes them from the cooling box. At one center where there was only one health worker, number tags were given to caretakers in the order they arrived, the health worker asked about ten caretakers at a time to sit on a circle of benches next to him, took their cards and filled in information about vaccine and next visit, asked caretakers prepare their childrens’ skin and administered vaccination. This process was the fastest of the once observed, though were children not weighed, health education only given very shortly and to a fraction of caretakers and only documentation reduced to the minimum. The overall impression was that the service session was highly impacted of the number of children receiving services. The health workers had more time give individualized health education when they had fewer children to immunize.
