# Supplementary material for: Assessment of a novel scanner-supported system for processing of child health and immunization data in Uganda
Source: BMC Health Serv Res. 2020 Apr 29;20:367. doi: 10.1186/s12913-020-05242-1 (PMC7191783; doi:10.1186/s12913-020-05242-1)
Supplement: Supplementary file 4 — Additional file 4. Code scheme for interview analysis. Interview results are summarized in a table including all sub-categories and codes. [file 12913_2020_5242_MOESM4_ESM.docx]

**Code scheme for interview analysis**
